# Peer review of "Behavioral Effects of Buspirone in Juvenile Zebrafish of Two Different Genetic Backgrounds"

_toxics, 2022, doi:10.3390/toxics10010022_

Round 1

Reviewer 1 Report

This manuscript shows the effect of buspirone, a drug used clinically to treat anxiety. The results of this work demonstrate how buspirone alters the locomotor behavior of zebrafish. The authors propose using behavioral analysis of juvenile zebrafish to study effects of anxiety treatments. The manuscript is well presented and very clear. My main concern with this work has been mentioned by the authors in the manuscript: the difficulty of attributing the effect on locomotion to changes in levels of anxiety. In my opinion the authors are honest in the manuscript and clearly reflect the limitations of their observations. I miss other possible explanations to the observed effects, for example, the effect may be due to a direct alteration of the locomotor system and not related to anxiety. Zebrafish have serotonergic neurons in the spinal cord that could be responsible for changes in behaviour or, buspirone could be affecting the activity of motor neurons by serotonin 1A receptor (see doi: 10.1002/dneu.22606).

Despite the fact that with the results presented here, changes in fish behavior cannot be related to anxiety, if the usefulness of zebrafish as a model and how interesting and accessible it is to study different aspects of their behavior to study the effect of different drugs. Furthermore, this manuscript may be the beginning of future studies that try to better define the mechanisms by which buspirone modulate the behavior of zebrafish.

The manuscript is well written, and the results are clear. Where I see that it could be improved is in the discussion. The authors mention the weak point of the study, that the results cannot be associated with anxiety but when they discuss it, they only focus on explaining this possibility without mentioning other mechanisms that could cause the results presented:

In the paragraph between the lines 431-463 they explain that the observed results cannot be attributed to the reduction in anxiety of the fish due to the treatment but that it is a possible explanation. In my opinion, it is well justified but the work would be better if they developed, briefly, other possible explanations for the results, which is what should be done in the discussion. For example, zebrafish have serotonergic neurons in the spinal cord and motor neurons are known to have serotonergic contacts (doi: 10.1076 / ejom.34.2.65.13021.). In addition, there are papers in which it is demonstrated how serotonin reduces the locomotion of zebrafish, similar results to those of this manuscript (doi: 10.1002 / dneu.22606; doi: 10.1523 / JNEUROSCI.1978-09.2009; doi: 10.1002 / neu .10292.). They should also refer to similar works that used drugs that modulate the serotonergic system and observed changes in the locomotor behavior of zebrafish (doi: 10.1016 / j.ntt.2007.07.005.). 

Author Response

Comments and Suggestions for Authors

This manuscript shows the effect of buspirone, a drug used clinically to treat anxiety. The results of this work demonstrate how buspirone alters the locomotor behavior of zebrafish. The authors propose using behavioral analysis of juvenile zebrafish to study effects of anxiety treatments. The manuscript is well presented and very clear. My main concern with this work has been mentioned by the authors in the manuscript: the difficulty of attributing the effect on locomotion to changes in levels of anxiety. In my opinion the authors are honest in the manuscript and clearly reflect the limitations of their observations. I miss other possible explanations to the observed effects, for example, the effect may be due to a direct alteration of the locomotor system and not related to anxiety. Zebrafish have serotonergic neurons in the spinal cord that could be responsible for changes in behaviour or, buspirone could be affecting the activity of motor neurons by serotonin 1A receptor (see doi: 10.1002/dneu.22606).

********************

We appreciate the praising comments, thank you.  Also, we completely agree with the referee with regard to potential alternative possibilities that may explain the observed buspirone effects.  We now mention the additional alternative the referee brought up, and also cite the paper he/she suggested.

********************

Despite the fact that with the results presented here, changes in fish behavior cannot be related to anxiety, if the usefulness of zebrafish as a model and how interesting and accessible it is to study different aspects of their behavior to study the effect of different drugs. Furthermore, this manuscript may be the beginning of future studies that try to better define the mechanisms by which buspirone modulate the behavior of zebrafish.

*****************

Again, we truly appreciate the praising comments.  And indeed, we view this study as only a first step on the long road ahead.  We emphasize in the manuscript that relatively little is known about the behavioural responses of the zebrafish, especially juvenile/larval zebrafish, thus we wanted to be careful with the interpretation of our behavioural findings.

******************

The manuscript is well written, and the results are clear. Where I see that it could be improved is in the discussion. The authors mention the weak point of the study, that the results cannot be associated with anxiety but when they discuss it, they only focus on explaining this possibility without mentioning other mechanisms that could cause the results presented:

In the paragraph between the lines 431-463 they explain that the observed results cannot be attributed to the reduction in anxiety of the fish due to the treatment but that it is a possible explanation. In my opinion, it is well justified but the work would be better if they developed, briefly, other possible explanations for the results, which is what should be done in the discussion. For example, zebrafish have serotonergic neurons in the spinal cord and motor neurons are known to have serotonergic contacts (doi: 10.1076 / ejom.34.2.65.13021.). In addition, there are papers in which it is demonstrated how serotonin reduces the locomotion of zebrafish, similar results to those of this manuscript (doi: 10.1002 / dneu.22606; doi: 10.1523 / JNEUROSCI.1978-09.2009; doi: 10.1002 / neu .10292.). They should also refer to similar works that used drugs that modulate the serotonergic system and observed changes in the locomotor behavior of zebrafish (doi: 10.1016 / j.ntt.2007.07.005.). 

***********************

We fully agree, and now we have expanded the discussion as suggested, for example, we briefly describe and cite the above suggested studies.

***********************

Reviewer 2 Report

General comments: Anxiety disorder is one of the most common mental diseases. Environmental strain can lead to such diseases and cause behavioral disorders. Zebrafish is one of most popular model organisms and was used to explore the influence of anxiety on behavior by the authors. By measuring the behavioral changes, this research provided evidence that zebrafish can be appropriate species to quantify effects of human anxiolytic drugs.

The specific comments were as follows:

  1. Line 46: “have been successfully employed in mutation as well as drug screens”-It would be more appropriate to change “have” to “has”.
  2. The authors set the exposure time to 1 hour as acute effects of buspirone and expected that the concentration of buspirone would reach a steady maximum level in equilibrium with that of the external bath in the brain of fish after 1 hour. Often, 24 hours or 96 hours are set as the acute exposure time and it may take more time for drugs to reach an equilibrium between the environment and organisms. Why did the author only set the exposure time to 1 hour?
  3. Line 504: “Gelfuso ÉA, Rosa DS, Fachin AL, Mortari MR, Cunha AO,”-the“,” should be revised to “;” like other references. Also, reference 1 (line 500), reference 5 (line 508,) reference 8 (514) the format should be consistent as for “&” in these references.
  4. The experiment was designed with buspirone concentration having 4 levels-how did the author determine to set these concentrations and the buspirone used in the experiment was crushed, homogenized, filtered, is it necessary to quantify the concentrations after the experiment with drug absorption, distribution, metabolism and excretion (ADME) related processes?
  5. Line 414 and line 416: “80 mg/liter” and “5mg/liter”. Please keep the format consistent - determine if spaces are required between numbers and units.
  6. Line 169: “27 oC” should be revised to 27 ℃.
  7. Line 171 “stage 6 days-post-fertilization” should be revised to “stage of 6 days-post-fertilization”.
  8. Line 179 and line 269 “mg/L” and “mg/liter”- keep the unit consistent.

Line 373: “effect of buspirone” should be revised to “effects of buspirone”.

Author Response

Comments and Suggestions for Authors

General comments: Anxiety disorder is one of the most common mental diseases. Environmental strain can lead to such diseases and cause behavioral disorders. Zebrafish is one of most popular model organisms and was used to explore the influence of anxiety on behavior by the authors. By measuring the behavioral changes, this research provided evidence that zebrafish can be appropriate species to quantify effects of human anxiolytic drugs.

The specific comments were as follows:

  1. Line 46: “have been successfully employed in mutation as well as drug screens”-It would be more appropriate to change “have” to “has”.

****************

Corrected

***************

  1. The authors set the exposure time to 1 hour as acute effects of buspirone and expected that the concentration of buspirone would reach a steady maximum level in equilibrium with that of the external bath in the brain of fish after 1 hour. Often, 24 hours or 96 hours are set as the acute exposure time and it may take more time for drugs to reach an equilibrium between the environment and organisms. Why did the author only set the exposure time to 1 hour?

**************

Several psychopharmacology studies use 30 min acute exposure periods, some use as short as 3 min in adult zebrafish (but see our comment to the question of ref 3 about whether we think such a short period is appropriate).  In juveniles/larval zebrafish, even shorter than 30 min exposure periods may be appropriate for testing acute drug effects (larger body surface/volume ratio, hence faster absorption).  We decided to employ 60 min exposure length because, in our hands, within 30 min most drugs, including buspirone, exert their maximum level behavioural effects, i.e., the effect subsequently plateaus.  Although, as we explicitly state in the manuscript, detailed ADME characterization of most drugs and compounds is lacking in zebrafish, the above suggests that 60 min immersion period is sufficient especially in juvenile zebrafish.  Please also note that the 60 min is only the pre-behaviour test immersion period, as this period is immediately followed by a subsequent 30 min immersion period during which the fish’s behaviour is quantified.  The lack of consistent temporal trajectories during this recording period also confirm our prior findings of a plateaud drug effect.  We now clarify the above in the revised manuscript.

*******************

  1. Line 504: “Gelfuso ÉA, Rosa DS, Fachin AL, Mortari MR, Cunha AO,”-the“,” should be revised to “;” like other references. Also, reference 1 (line 500), reference 5 (line 508,) reference 8 (514) the format should be consistent as for “&” in these references.

**************

Corrected

*************

  1. The experiment was designed with buspirone concentration having 4 levels-how did the author determine to set these concentrations and the buspirone used in the experiment was crushed, homogenized, filtered, is it necessary to quantify the concentrations after the experiment with drug absorption, distribution, metabolism and excretion (ADME) related processes?

*****************

Thank you for spotting our omission.  We now explain the following: “The buspirone concentrations were selected based upon prior studies with zebrafish [21, 49, 50, 53] and also upon our own preliminary studies showing no side effects, mortality or morbidity even at the highest dose chosen.”

******************

  1. Line 414 and line 416: “80 mg/liter” and “5mg/liter”. Please keep the format consistent - determine if spaces are required between numbers and units.

*********************

Corrected

**************

  1. Line 169: “27 oC” should be revised to 27 ℃.

******************

Corrected

*******************

  1. Line 171 “stage 6 days-post-fertilization” should be revised to “stage of 6 days-post-fertilization”.

*******************

Corrected to: “……stage at 6 days-post-fertilization”

*********************

  1. Line 179 and line 269 “mg/L” and “mg/liter”- keep the unit consistent.

*******************

Corrected, unit is now consistently referred to as mg/l

*******************

Line 373: “effect of buspirone” should be revised to “effects of buspirone”.

*******************

Corrected

*******************

Reviewer 3 Report

In the manuscript toxic-1514141 by Abozaid and Gerlai, authors examined the anxiolytic effects of Buspirone in juvenile zebrafish from two different background.

Anxiety and fear responses are not well characterized in the juvenile zebrafish. So, use of juvenile zebrafish as sensitive model for anxiolytic drugs is not a good idea. Without confirming that these phenotypes/features are readout of anxiety or are due to anxiety there is no point of highlighting that sensitivity against anxiolytic drug was examined.

As compared to the previously published studies this study does not provide any addition to the existing knowledge.

I have following comments:

  1. Title is not conclusive one. It misleads that genetic background did have any effect, while it is not the case.

  1. Authors names should be check for the correctness.

  1. Abstract does not provide the clear question of the study? In other words, what is not known either effectiveness of drugs or appropriate model?

  1. What was the basis of choosing the dose range of buspirone? Similar for the exposure time?

  1. Introduction section does not explain well what is known about the anxiety/fear response and what is not? Line 55 states a very wage sentence with out explanation.

  1. Line 62. what is the basis for referring these aversive reactions as anxiety response?
  2. At the conclusion, authors themselves mentioned that it is not clear if these responses are reduced anxiety related.

  1. Line 100-101. Please explain this. The way paragraph is ended, it indicates that authors aim to examine this in the presented study.

  1. Line 411-414. Not all parameters appear to be linearly dose dependent. Did author performed correlation regression analysis to conclude this?

  1. Use of an earlier stage of development and comparatively longer exposure time.
  2. In previous study (Bencan et al., 2009), Zebrafish were examined against exposure to Buspirone concentrations 0-50 mg/L) for 3 min.

  1. Most of the studied parameters showed dose dependent response against high concentrations 20 or 80mg/L.

  1. Some responses are inconsistent with anxiolytic response of buspirone. It could be adverse effect of exposure to high dose.

  1. Line 482-486. It is not appropriate to conclude based on data obtained from the used model against high dose and long exposure.

  1. Manuscript needs a through revision for syntax errors and typo.

Author Response

Comments and Suggestions for Authors

In the manuscript toxic-1514141 by Abozaid and Gerlai, authors examined the anxiolytic effects of Buspirone in juvenile zebrafish from two different background.

Anxiety and fear responses are not well characterized in the juvenile zebrafish. So, use of juvenile zebrafish as sensitive model for anxiolytic drugs is not a good idea. Without confirming that these phenotypes/features are readout of anxiety or are due to anxiety there is no point of highlighting that sensitivity against anxiolytic drug was examined.

****************

The referee misses numerous points about why the zebrafish juvenile may be an excellent subject for testing effects of drugs and compounds with potential anxiety altering properties.  Just because research on anxiety-like responses is in its infancy for juvenile zebrafish, one should not say that such research has no merit.  Exactly because of the paucity of our knowledge in this area, one should do such research.  The referee thinks that only well validated animal models are useful for such research.  But the opposite is true.  Well validated drugs, having known anxiolytic properties like buspirone should be used to test how novel, cheaper and potentially more efficient model organisms, such as the zebrafish, may respond.  This is explicitly explained in the manuscript at multiple places, including the introduction and discussion.

****************

As compared to the previously published studies this study does not provide any addition to the existing knowledge.

**********************

This is a surprising statement from the referee, given that he/she just explained above that our understanding of zebrafish anxiety and how this species responds to anxiolytic drugs is minimal.  Our study is aimed at increasing our knowledge in this very area, and thus we believe the referee is incorrect, and certainly inconsistent in his/her opinion.

*******************

I have following comments:

  1. Title is not conclusive one. It misleads that genetic background did have any effect, while it is not the case.

*****************

We disagree.  The title is descriptive, and precisely states what this study is about: identification and description of “Behavioural effects of buspirone in juvenile zebrafish of two different genetic backgrounds”  The title neither states nor implies genetic background effects.  It simply states that two genetic backgrounds were investigated.

*******************

  1. Authors names should be check for the correctness.

******************

Author names have been checked.  So is English grammar, unlike in the above point made by the referee.

******************

  1. Abstract does not provide the clear question of the study? In other words, what is not known either effectiveness of drugs or appropriate model?

*****************

We disagree.  The Abstract precisely describes the rationale for, and the findings and conclusions of this study.

*****************

  1. What was the basis of choosing the dose range of buspirone? Similar for the exposure time?

******************

Please see answers to the other referees’ questions above.

*****************

  1. Introduction section does not explain well what is known about the anxiety/fear response and what is not? Line 55 states a very wage sentence with out explanation.

********************

We again disagree.  In fact, we think the introduction provides quite an extensive context for our study.  It is more extensive than most empirical papers published in Toxics and other peer reviewed journals.  For example, it incorporates and discusses results and conclusions published in 96 peer reviewed publications, a number that is on par with how many papers are cited in comprehensive review articles.  For specific details as to the question about fear/anxiety, please read the Introduction and Discussion sections.  

We do not understand what the referee means.  What is a “very wage sentence”?  And what does “with out” mean?  The sentence we wrote in the manuscript the referee is referring to is as follows: “Last, the effects of mutations and drugs can be efficiently detected by the growing number of behavioural test paradigms developed for this species [19,31-35].”  We do not wish to elaborate on this topic further, as the cited papers do this, and there is no need to say anything else.

*****************

  1. Line 62. what is the basis for referring these aversive reactions as anxiety response?

******************

We do not understand why this is question given that the paragraph immediately following line 62 answers it in a detailed manner with relevant studies cited and explained there.

*****************

  1. At the conclusion, authors themselves mentioned that it is not clear if these responses are reduced anxiety related.

*********************

That is correct.

********************

  1. Line 100-101. Please explain this. The way paragraph is ended, it indicates that authors aim to examine this in the presented study.

*******************

Lines 100 and 101 read as follows: “…be an antagonist for the dopamine D2 autoreceptor in mammals, which inhibits dopaminergic neurotransmission [48].  In zebrafish, its psychopharmacological profile or..”  we have no idea what explanation the referee needs.

***************

  1. Line 411-414. Not all parameters appear to be linearly dose dependent. Did author performed correlation regression analysis to conclude this?

********************

Again, we do not understand why the referee is asking this.  Linearity is not statistically tested in our study.  We simply refer to the pattern of dose responses when we say linear or qusi-linear.  This is common practice in psychopharmacology.  Also, what is a “correlation regression analysis”?  It doesn’t exist!  Besides, Pearson product moment or Spearman rank correlation-based correlation coefficients will not tell linearity or non-linearity of dose responses apart as both assume linearity and do not distinguish these two possibilities.  Same is true for regression analysis.

*******************

  1. Use of an earlier stage of development and comparatively longer exposure time.

*****************

Again, we do not understand what the referee is saying.  Is he/she saying we should use younger zebrafish and longer exposure periods?  Why?  Is there evidence that buspirone is ineffective at the developmental stage of zebrafish when we tested its effects?  Is there any evidence that a 60 min long acute exposure period is ineffective or too long?  We have already cited several papers that clearly show the answer to these questions is no.  Please peruse our introduction and perhaps also the discussion sections.

*****************

  1. In previous study (Bencan et al., 2009), Zebrafish were examined against exposure to Buspirone concentrations 0-50 mg/L) for 3 min.

*********************

We are aware of the period of immersion employed by Bencan et al., 2009.  We think these authors made a mistake.  This exposure period is too short for adult zebrafish, and likely for larval/juvenile zebrafish too, especially given that we have no ADME characterization of buspirone in zebrafish.  The problem with too short exposure periods is that they bias the drug effects to peripheral sensory effects as opposed to central nervous system effects or other systemic effects.  Please also note that other refs are asking why we used only 60 min long exposure and longer, perhaps several hours longer, periods.  If in doubt, please read our answers to this question above as well as the justification for the chosen exposure length described in the revised manuscript.

*********************

  1. Most of the studied parameters showed dose dependent response against high concentrations 20 or 80mg/L.

*********************

Yes, but also observe the quasi-linear dose response curves for several behaviours.  This is exactly what one expects in case of a correctly chosen dose series.  For further info, please consult pharmacology or psychopharmacology textbooks.

*********************

  1. Some responses are inconsistent with anxiolytic response of buspirone. It could be adverse effect of exposure to high dose.

*********************

Thank you for asking this important question.  We observed no adverse effects, grossly abnormal motor or posture patterns, morbidity or mortality.  This is now clarified in the revised manuscript.  Also see above response regarding the quasi-linearity of dose responses.

And yes, some of the responses do not appear to mimic what we know about anxiety in adult zebrafish.  But, as we discussed in our paper, we do not yet fully understand the fear and anxiety repertoire of adult zebrafish and even less we understand that of juvenile/larval zebrafish.  We discuss this in the context of what we know.  And what we know is that fear and anxiety responses are environmental context and stimulus specific in adult zebrafish.  We discuss this question also in the context of active versus passive avoidance reactions.  Thus, the assertion that we know what to expect of an anxious juvenile zebrafish is wrong.  So is the assertion that some of the currently observed buspirone effects show alterations clearly inconsistent with anxiolytic response repertoires.  Again, this is a matter of proper interpretation of results and not jumping to conclusions too fast, exactly because we do not know enough.  We attempt to explicitly clarify these points in our discussion section.

*********************

  1. Line 482-486. It is not appropriate to conclude based on data obtained from the used model against high dose and long exposure.

***************

Dose is not too high and exposure length is not too long based upon findings in the literature and on our preliminary studies, please see the literature we cited if in doubt.

***************

  1. Manuscript needs a through revision for syntax errors and typo.

****************

While we did correct a few errors, including punctuation, inconsistent use of “and” or “&”, or inconsistent use of “l”, “L” versus “liter”, for example, in the revised manuscript, we believe the referee’s above assertion is a gross overstatement.  It is ironic that just in the short sentence with which he/she explains we need a thorough revision, he/she makes errors, e.g. writing “through revision”, (thorough is what he/she meant) and “typo”, instead of “typos”.

*****************

Reviewer 4 Report

Manuscript ID: toxics-1514141

Title: Behavioural effects of buspirone in juvenile zebrafish of two different genetic backgrounds

General Comments:

This manuscript describes the impact of buspirone exposure, an anxiolytic medication, on the behavioral responses of 2 different zebrafish populations. The paper is very well written and easy to follow, and the topic covered is an appropriate fit to the journal. I have 2 major concerns with this manuscript that should be addressed prior to publication.

1.The biggest short coming of the study is the exposure concentrations are nominal and not analytically validated, which limits the quality of the data and the reproducibility of these results. The authors would significantly improve the study by including analytical validation of their exposure concentrations.

  1. Could decreased activity be due to toxicity as opposed to just anti-anxiety response? A lot of the behavioral responses observed by the authors also coincide with the behavior of fish impaired due to toxicity. How do the authors make this distinction? Were any mortalities observed after exposure? Was there any physiological data to determine that this exposure was truly sublethal? More information is needed.

Specific Comments:

Abstract – should include some reference to the similarity and/or differences in responses between the two zebrafish strains, as that is one of the more interesting endpoints in the study

Line 175 – The animal use protocol number for the study should be referenced in this section.

Author Response

Comments and Suggestions for Authors

Manuscript ID: toxics-1514141

Title: Behavioural effects of buspirone in juvenile zebrafish of two different genetic backgrounds

General Comments:

This manuscript describes the impact of buspirone exposure, an anxiolytic medication, on the behavioral responses of 2 different zebrafish populations. The paper is very well written and easy to follow, and the topic covered is an appropriate fit to the journal. I have 2 major concerns with this manuscript that should be addressed prior to publication.

**************

Thank you for the praising comments.

***************

1.The biggest short coming of the study is the exposure concentrations are nominal and not analytically validated, which limits the quality of the data and the reproducibility of these results. The authors would significantly improve the study by including analytical validation of their exposure concentrations.

****************

This is an excellent point and we fully agree.  Nevertheless, we will not be able to provide analytical validation. That is, we will not be able to give detailed ADME characterisation of buspirone in the juvenile zebrafish.  This is simply because we are not set up to conduct such studies.  However, we also note that such characterization, for example establishing the precise amount of buspirone in different tissues, characterizing the time course of buspirone concentration changes in the brain and blood, running receptor occupancy studies, would be a substantial undertaking for even those who are experts in such methods.  This is simply due to the small size of the zebrafish and the fact that such studies have not been routinely conducted with this model system.  Briefly, it would take a lot of work to establish these analytical methods.  Nevertheless, we agree that this is a significant shortcoming of the zebrafish field, a major drawback that currently limits the utility of this species.  On the other hand, we view behavioural phenotypes as a quick and dirty proxy to such analytical methods.  This is why we regard our study only as a proof of concept work.  Proof of the concept that juvenile zebrafish may be a good tool with which effects of anxiolytic drugs/compounds may be investigated. 

*****************

  1. Could decreased activity be due to toxicity as opposed to just anti-anxiety response? A lot of the behavioral responses observed by the authors also coincide with the behavior of fish impaired due to toxicity. How do the authors make this distinction? Were any mortalities observed after exposure? Was there any physiological data to determine that this exposure was truly sublethal? More information is needed.

**************

Again, we agree.  We recognize the importance of making the distinction between toxicity and central nervous system related changes like anxiety.  However, at this point, all we can say is that at the concentrations employed, mortality and obvious signs of impairments were not detected.  To us, however, the borderline between toxicity and behavioural effects is not a sharp well-defined line, but a zone with shades of grey as opposed to black or white.  We now briefly discuss these questions in the revised manuscript as we recognize the importance of considering numerous alternative hypotheses.

**************

Specific Comments:

Abstract – should include some reference to the similarity and/or differences in responses between the two zebrafish strains, as that is one of the more interesting endpoints in the study

********************

We agree and state the following “Buspirone decreased distance moved, number of immobility episodes and thigmotaxis, and it increased immobility duration and turn angle in a quasi-liner dose dependent but genotype independent manner.”

******************

Line 175 – The animal use protocol number for the study should be referenced in this section.

******************

Information added as requested

***************

Round 2

Reviewer 3 Report

Recommend acceptance in present form.

Author Response

Thank you!

Reviewer 4 Report

All my comments have been addressed, no further concerns.

Author Response

Thank you!